# Efficacy of CMX001 as a Prophylactic and Presymptomatic Antiviral Agent in New Zealand White Rabbits Infected with Rabbitpox Virus, a Model for Orthopoxvirus Infections of Humans

**DOI:** 10.3390/v3020063

**Published:** 2011-01-25

**Authors:** Amanda D. Rice, Mathew M. Adams, Bernhard Lampert, Scott Foster, Randall Lanier, Alice Robertson, George Painter, Richard W. Moyer

**Affiliations:** 1 Department of Molecular Genetics and Microbiology, University of Florida, 1600 SW Archer Rd, Gainesville, FL 32610, USA; E-Mails: amandar@ufl.edu (A.D.R.); mathew.adams@csiro.au (M.M.A.); 2 Chimerix, Inc., 2505 Meridian Parkway Suite, 340 Durham, NC 27713, USA; E-Mails: blampert@chimerix.com (B.L.); sfoster@chimerix.com (S.F.); rlanier@chimerix.com (R.L.); arobertson@chimerix.com (A.R.); gpainter@chimerix.com (G.P.)

**Keywords:** CMX001, poxvirus, smallpox treatment, antiviral, rabbitpox

## Abstract

CMX001, a lipophilic nucleotide analog formed by covalently linking 3-(hexdecyloxy)propan-1-ol to cidofovir (CDV), is being developed as a treatment for smallpox. CMX001 has dramatically increased potency *versus* CDV against all dsDNA viruses and, in contrast to CDV, is orally available and has shown no evidence of nephrotoxicity in healthy volunteers or severely ill transplant patients to date. Although smallpox has been eliminated from the environment, treatments are urgently being sought due to the risk of smallpox being used as a bioterrorism agent and for monkeypox virus, a zoonotic disease of Africa, and adverse reactions to smallpox virus vaccinations. In the absence of human cases of smallpox, new treatments must be tested for efficacy in animal models. Here we first review and discuss the rabbitpox virus (RPV) infection of New Zealand White rabbits as a model for smallpox to test the efficacy of CMX001 as a prophylactic and early disease antiviral. Our results should also be applicable to monkeypox virus infections and for treatment of adverse reactions to smallpox vaccination.

## 1. Introduction

Although variola virus—the causative agent of human smallpox—has been eliminated from the natural environment, a human threat from weaponized variola virus remains [1–3]. Studies of variola virus antiviral compounds must therefore rely on testing in small animal models in order to evaluate potential efficacy [4]. In this study, we have used New Zealand White rabbits intradermally infected with rabbitpox virus strain Utrecht (RPV) as a model for smallpox and for human cases of monkeypox virus. Briefly, the characteristics that make RPV extremely attractive as a surrogate of variola and monkeypox viruses include: genetic similarity to both variola and monkeypox viruses, similarity of the pathophysiologic course of disease, low dose of virus to create lethal disease, correlation of viral burden with disease progression, and potential to model lesional disease. RPV virus, which causes a severe and lethal disease in rabbits, was first identified in the 1940s following an outbreak in a laboratory rabbit colony in Utrecht, Netherlands [5,6]. RPV and variola virus belong to the genus Orthopoxvirus, a group of viruses which share serological cross reactivity, genomic structure, virion morphology, and replication strategy [7–10]. Orthopoxviruses share similar pathogenesis in susceptible hosts, although some orthopoxviruses, such as variola virus, are very host restricted [11–14]. Interaction between the virus and host immune responses are thought to underlie the host range [15]. RPV is most closely related to vaccinia virus and is usually categorized as a subspecies of vaccinia (VV) [16], a classification substantiated by comparison of genomic sequences, which show an overall sequence similarity of 95% at the amino acid level [17]. RPV has enhanced pathogenicity for rabbits compared with VV and can be naturally transmitted in confined populations of rabbits, resulting in rapid spread and high mortality [18]. Transmission occurs via naturally generated aerosols in a manner similar to smallpox [18–20]. The ability of RPV to be easily spread by aerosol to uninfected rabbits and the overall course of disease closely parallels variola infections of human populations [21].

Intradermal inoculation of RPV in rabbits is a useful and relevant route of infection to model smallpox or monkeypox because a known inoculum volume can be delivered precisely and the course of disease is virtually indistinguishable from aerosol mediated rabbit to rabbit infection [19,22,23]. While the disease progression of intradermal and aerosol infections are clinically similar, it is important to note several differences between the two routes of infection, each offering specific key animal model advantages for human smallpox. First, the aerosol route of infection requires twice as much virus required to reach the LD_50_ when compared to intradermally infected animals, *i.e.*, (20 pfu for aerosol [22,23] *vs.* 10 pfu for intradermal). The disease symptoms requiring euthanasia in both routes is respiratory distress, however this occurs 1–2 days sooner in aerosol infected animals than in intradermally infected rabbits, most likely due to differences in the primary site of infection (RPV intradermally inoculated must first replicate at the flank, spread to the lungs and cause pathology while RPV introduced via the aerosol route is instilled directly into the lung and does not need not to spread to cause respiratory distress). RPV intradermally inoculated into rabbits at very low doses (100 pfu) causes a systemic disease, extensive viremia, the development of secondary lesions, subsequent severe respiratory disease and death by nine days post infection. Thus, RPV infection of rabbits is a highly sensitive model for orthopoxvirus disease as well as spread and can be used to evaluate efficacy of poxvirus vaccines and antivirals.

CMX001 is a lipophilic nucleotide analog formed by covalently linking 3-(hexdecyloxy)propan-1-ol to cidofovir (CDV). Cidofovir (marketed as Vistide^®^), an antiviral drug approved for the treatment of CMV-retinitis [24,25], is an alternative substrate inhibitor of the DNA polymerases encoded by orthopoxviruses, and has shown activity in lethal models of poxvirus infection using mice and monkeys [26–30]. Although CDV is the only antiviral drug currently available for use in the event of a smallpox outbreak, its utility in an attack would be limited since it must be administered by slow intravenous infusion and has the potential for significant nephrotoxicity [31,32]. In an effort to address the need for an orally available antiviral drug for human poxvirus infections, a lipid conjugate of CDV was synthesized by covalently coupling CDV to hexadecylpropanediol, the resulting compound is referred to as CMX001 here forth. The conjugate was designed to resemble a natural phospholipid and utilize natural uptake pathways to achieve oral availability, high uptake in target cells and overall improved Absorption, Distribution, Metabolism, Excretion (ADME) profiles [33].

In addition to the overall genetic similarity among orthopoxviruses, including variola and monkeypox viruses, the virally encoded DNA polymerase, which is the target of antiviral action for the active metabolite of CMX001, shows 97.9% amino acid identity and 99.2% strong similarity between RPV and a consensus of 48 variola virus isolates [34]. *In vitro* studies have shown that CMX001 is active against both RPV and variola viruses with IC_50_ values of 0.5 μM and 0.1 μM, respectively [35]. CMX001 has been previously shown to be effective *in vivo* using murine models of orthopoxvirus infections [36–38].

We have used the intradermal inoculation RPV model to evaluate the efficacy of CMX001 as a potential prophylactic treatment for orthopoxvirus infection [19]. In this communication, our results demonstrate that CMX001 is effective in preventing mortality and reducing morbidity when administered prior to virus exposure and early in infection as compared to untreated animals.

## 2. Results and Discussion

### 2.1. The Infection of Rabbits by Rabbitpox Virus: Model Review

The standardization of the rabbit/RPV model has been previously published [19] by our laboratory and the timing of both clinical symptoms and euthanasia criteria appearance are shown in Figure 1. The model is reviewed and summarized here to highlight the features important to evaluating antiviral compounds. Only the features of smallpox and rabbitpox virus infections are compared but monkeypox virus infections share similar properties. This model, which uses New Zealand White (NZW) rabbits infected with 100–1000 pfu of RPV (administered bilaterally) by intradermal injection, if untreated, is almost uniformly lethal (Figure 2A). There are few differences between 100 and 1000 pfu observed in RPV infected nine week old NZW rabbits most likely due to the fact the doses are 10× and 100×, respectively, the LD_50_ dose. The appearance of swelling at the primary site of infection occurs 12–24 hours earlier in animals infected with 1000 pfu as compared to 100 pfu, but the overall symptomology follows the same time line. The disease begins with a local reaction at the site of virus introduction visualized as a raised red swelling within 1–2 days post infection (dpi). The lesion continues to grow in both diameter and thickness over the next 6–8 days. Necrosis at the primary site of infection develops by 3 to 4 dpi. The lesion reaches maximal diameter of 8–10 cm, with a thickness of 1–2 cm and encompasses the entire flank of the rabbit at the time of euthanasia. The progression of the primary lesion over time is shown in Figure 3.

The first clinical signs of systemic disease are the presentation of a fever (Figure 2C) and failure to gain weight (Figure 2B) that begins at 3–4 dpi. Weight loss continued until the animals are euthanized. A failure to gain weight for young, growing animals is a sign of disease and weight loss is a symptom of severe disease. A fever in the rabbits is a body temperature of 39.5°C or higher as measured by the subcutaneous temperature transponders. RPV infected animals exhibited fevers generally from 3 dpi to euthanasia when the body temperatures of infected animals could on occasion drop drastically. The severity of disease is quantified using the clinical score (Figure 2D); RPV infected animals exhibited severe disease with an average maximum clinical score of 22.4 points out of a possible 33 points.

Secondary lesions were initially observed in the ears and generally appear 3–5 dpi, 12–24 hours after the first sign of a fever is observed. The lesions first appeared in the ears as small red spots typically found at the bend of a blood vessel and are easily visualized by backlighting the ears. The progression of these small red spots to pustular lesions on the ears is shown in Figure 3. It is important to note that the secondary lesions found in the ears, unlike those observed with vaccinia virus, are small because the rabbits succumb to disease before the lesions fully develop. Secondary lesions were also found on the nose, eyelids, in and around the mouth, genitals and as a rash across the body of the rabbit.

Animals infected with RPV were ultimately euthanized due to respiratory distress characterized by open mouth breathing, a decrease in respiration rate to below 40 breaths per minute or severe lung sounds. The respiratory symptoms began at approximately 5 dpi and progress to euthanasia criteria levels by 7–9 dpi. At the time of euthanasia it was typical to observe profuse mucopurulent discharge from the nostrils and severe discharge from the eyes.

Upon necropsy, virus was recovered from all tissues collected including the skin (both lesion and non-lesion containing normal appearing skin), lungs, liver, spleen, gonad and blood. The LD_50_ of RPV in nine week old 3–4 lb NZW rabbits is approximately 10 pfu (data not shown).

RPV in rabbits shares many of the same pathophysiologic features of variola in humans. The replication and spread of RPV and variola within the host are believed to be very similar [15]. In both RPV and smallpox, there is an incubation period during which the virus replicates locally and spreads to key tissues, most notably the lymphoid cells of the reticuloendothelial system. Replication in these cells produces a secondary viremia which results in disseminated infection affecting a wide range of organs. Both RPV and smallpox produce fever, malaise, and the typical pox lesions on the skin. In addition, both RPV and smallpox exhibit high mortality rates [15,20]. Monkeypox in humans results in a very similar disease although lethality is lower.

The occurrence of lesions in the RPV model is an important parallel with smallpox infections. Human data with smallpox show a positive relationship between the extent of lesions and the mortality rate. For ordinary-type variola major infections, increased severity of lesions—discrete, semi-confluent, and confluent—was correlated with increased death rates of 9.3, 37, and 62%, respectively [15,39].

The degree of involvement of the respiratory tract is perhaps the most significant difference between RPV and smallpox. In RPV, the respiratory tract is a key site of viral replication whether the animals are infected via the respiratory tract, intradermally, or by animal to animal transmission [19,20]. Rabbits infected with RPV exhibit obvious signs of respiratory distress including profuse mucopurulent discharge from the nostrils and slow, labored, frequently open-mouth breathing [19]. Respiratory distress is the most frequent trigger for euthanasia in RPV [19]. In smallpox, respiratory distress is usually not cited as the cause of death; however, there is clearly respiratory tract involvement. For example, the earliest lesions in smallpox cases are those occurring in the oropharyngeal cavities which are thought to be the result of the secondary viremia [15]. Furthermore, in his review of literature, Martin [40] noted viral pneumonia and airway compromise as key contributing causes of smallpox-related death. Therefore, the difference in respiratory tract involvement between RPV and smallpox may be one of degree rather than an absolute difference.

Another key difference between RPV and smallpox is the time course of the disease. In smallpox, deaths occur approximately 22 to 28 days after infection, whereas in RPV, deaths (euthanasia) in vehicle treated intradermally infected animals occur between six and 10 days post infection. Therefore, the window of opportunity for effective antiviral therapy is expected to be larger in smallpox compared to RPV.

It is also informative to consider the cause of death in smallpox *versus* RPV. The cause of death in smallpox has been debated over the years, but one current view is that uncontrolled inflammatory responses resulting from a deregulated cytokine cascade contribute to a shock-like syndrome, hypotension, and failure of key organs [41,42]. As noted above, the most frequent trigger for euthanasia in RPV is respiratory distress. However in studies where rabbits were allowed to die naturally of the infection, extreme hypotension and elevated potassium levels were cited as the most consistent physiological changes [43,44]. In speculating on the cause of the hypotension, Boulter *et al.* comment that “it is more probable that the endogenous mechanisms activated in general inflammation ... are implicated.” Therefore, the underlying disease processes leading to death may be very similar between smallpox and RPV.

### 2.2. Efficacy of Prophylactic administration of CMX001 in Preventing RPV Induced Disease

The initial study performed was to evaluate and determine the minimum dose of CMX001 required to protect otherwise lethally infected rabbits from RPV disease. Animals received either 100 or 1000 pfu RPV. For this report, these animals were grouped together based upon dosage of CMX001 received. There was no difference between the groups that received 100 or 1000 pfu RPV in severity of disease presentation for the initial experiments; therefore, for all future studies 100 pfu RPV was used. It is noteworthy that although the clinical presentation was virtually identical, animals receiving the higher dose of RPV did exhibit clinical symptoms 12–24 hours earlier than those animals receiving 100 pfu RPV. CMX001 was administered prior to infection as a pre-exposure prophylactic treatment. Protective effects of CMX001 were evaluated at doses of 1, 5, 10 and 20 mg/kg. Animals that received dosages of 1, 5 or 10 mg/kg received CMX001 twice a day (BID), morning and late afternoon, for five days beginning one day prior to infection. The group of animals receiving 20 mg/kg were administered CMX001 once a day (QD) in the morning for five days beginning one day prior to infection. All tested doses of CMX001 were protective against lethal RPV disease (Table 1) while vehicle treated animals were euthanized at 6.6 ± 0.16 dpi due to respiratory distress.

Although all the animals that received CMX001 were protected from lethal RPV disease there was a difference in clinical symptoms that was dependent on the dose of CMX001 received. Animals treated with 5 or 10 mg/kg BID or 20 mg/kg QD exhibited few clinical symptoms over the course of the experiment. All three treatment groups exhibited a positive weight change, indicating a nearly constant weight gain, with no significant differences between groups (Figure 4A). Animals that received 1 mg/kg of CMX001 exhibited a weight loss profile similar to that of the vehicle treated animals (Figure 4A).

Animals treated with 1 mg/kg CMX001 also exhibited a body temperature increase similar to that in both severity and duration compared to the vehicle treated animals (Figure 4B). The 5 mg/kg treated group exhibited a mild temperature spike with a maximal temperature of 40.2 °C as compared to 41.2 °C for vehicle treated animals. The temperature spike was three days later than that observed with either the 1 mg/kg or the vehicle treated animals lasting for approximately the same duration of four days (Figure 4B).

While the animals exhibited both weight loss and fevers, the overall health of the animals treated with 1 mg/kg, as measured by the more comprehensive overall clinical score, was markedly lower than that of the vehicle treated animals (Figure 4C). The overall level of disease as measured by clinical scores demonstrated that animals treated with 5, 10 or 20 mg/kg CMX001 exhibited only mild disease (Figure 4C).

The primary lesions of animals treated with 1 mg/kg CMX001 did become swollen and necrotic but did not become as large or edematous as those of the vehicle treated animals (Figure 4D compare first and last photographs). Animals treated with 1 mg/kg CMX001 exhibited numerous secondary lesions on the ears, nose and mouths (data not shown). The primary lesions of animals treated with 5 mg/kg were small in diameter and exhibited necrosis early in the experiment that rapidly resolved to a scab. Animals treated with 10 or 20 mg/kg exhibited no noteworthy primary lesion with little to no swelling early and no necrosis (Figure 4D). These animals also exhibited few if any secondary lesions on the ears, nose or mouth as compared to vehicle or 1 mg/kg CMX001 treated animals (data not shown).

The clinical score is used as a more comprehensive and inclusive measure of overall health of the study animals and as such can be used to gauge the severity of disease observed throughout the course of the experiment. A concern with any antiviral treatment is the shifting or simply delaying the disease course corresponding to withdrawal of the antiviral agent. Therefore, to assess the level of protection CMX001 provided to treated animals, the maximum clinical score and maximum weight loss for each animal were compared.

When the maximum clinical score for a group over the course of the experiment is used as a measure of maximal illness observed during the course of the experiment it is observed that treatment with CMX001 provides a statistically significant reduction in the disease severity for all treatment groups (Figure 5A). While the maximum weight loss for each treatment group is not statistically significant when compared to the maximum weight loss of the vehicle treated animals, a dose dependent trend was observed (Figure 5B). Animals treated with 1 mg/kg CMX001 did not follow this trend, however, exhibited a larger weight loss than vehicle treated animals. This was attributed to a combination of multiple secondary lesions in the mouth causing difficulty for the animals to eat food and their prolonged survival as compared to vehicle animals.

Upon necropsy at euthanasia (14 dpi), no virus was detected in any of the sampled internal organs in any of the treated animals. Virus was only detected at the primary site of infection. In contrast, vehicle treated animals had virus detected in all tissues sampled as well as at the primary site of infection (data not shown).

### 2.3. CMX001 Dose Response against RPV Infection

Based upon results from the experiments to evaluate the efficacy of CMX001 when administered as a pre-exposure prophylactic, the next evaluation of CMX001 was to determine whether treatment once a day beginning just after exposure during the asymptomatic period would also be effective against RPV induced disease. We examined the ability of CMX001 to reduce morbidity and prevent mortality at different doses when treatment was initiated at Day 1 post infection where animals received only a single daily dose of CMX001. The doses used were 2, 5, 10, and 20 mg/kg once a day for five days beginning at 1 dpi. As shown in Table 2, no rabbits treated with CMX001 were euthanized for severe RPV disease during the 14-day study period while animals receiving vehicle required euthanasia at 7 ± 1 dpi.

Although all rabbits dosed with CMX001 survived, a dose dependent response in disease severity was observed between the treatment groups. The degree of weight loss and recovery from RPV disease measured by the return of weight gain was CMX001 dose dependent. Animals that received 2 mg/kg exhibited mild weight loss from 4 to 10 dpi while the weight loss exhibited by 5 mg/kg treated animals was similar in rate to the 2 mg/kg treatment group but returned to a positive weight gain by 8 dpi. Rabbits that received 10 or 20 mg/kg CMX001 showed few symptoms including no substantial weight loss with a general positive trend in weight change (Figure 6A). The maximum weight loss for each group was also evaluated (Figure 7B) and again demonstrated an inverse relationship between the dose of CMX001 and weight loss in which the higher doses of CMX001 demonstrated virtually no weight loss from baseline weight.

All treatment groups exhibited temperature spikes from 3 to 8 dpi with similar profiles, however there was a dose response trend observed in which the groups treated with lower concentrations of drug exhibited higher maximal temperature spikes (Figure 6B). The overall disease as measured by clinical scores showed the same trend observed with weight loss and body temperature in which there was a dose response where the higher doses of CMX001 led to a lower clinical score indicating fewer symptoms of RPV induced disease (Figure 6C). The primary lesions for all treated groups were similar as observed in the first study in which the higher the dosage of CMX001 the less swelling, edema and necrosis present (Figure 6D). Animals treated with 20 mg/kg had virtually no sign of a primary lesion by 5 dpi while animals treated with 5 or 10 mg/kg exhibited a small slightly swollen area with a central scab. Animals treated with 2 mg/kg CMX001 exhibited large primary lesions with necrosis that progressed to scabbing with the overall size of the lesions being larger in diameter from the other treatment groups. The presence of secondary lesions also inversely corresponded to the dose of CMX001 received in which animals that received 20 mg/kg CMX001 had no secondary lesions while those that received 2mg/kg had numerous secondary lesions.

Again, using the maximum clinical score and weight loss as indications of maximal illness it was observed that treatment with CMX001 provides a reduction in the disease severity for all treatment groups (Figure 7A). While the maximum weight loss for each treatment group is not statistically significant when compared to the −3.31% ± 0.79 weight loss maximum for vehicle treated animals, there is a dose dependent trend observed (Figure 7B). While the weight loss for animals treated with 2 and 5 mg/kg CMX001 is larger than that observed with vehicle treated animals, this may again be attributed to number of secondary lesions in the mouth and prolonged survival as compared to vehicle animals.

We found that the CMX001 given once a day from Day 1 to Day 5 PI protects rabbits from lethal disease caused by RPV virus. These animals fared much better than the previous QD group that began treatment at Day -1 instead of Day 1, since the current drug dosing plan afforded the rabbits two extra days of drug protection during the actual viral infection.

### 2.4. Comparison of CMX001 Treatment Time of Initiation

Given that CMX001 at relatively low doses was protective against RPV induced morbidity and mortality, we examined the impact of a delay in dosing on the development of severe disease. Therefore, we examined the ability of CMX001 to reduce morbidity and prevent mortality when administered at 5 mg/kg BID for five days when treatment was initiated at −1, 0, 1, 2, 3, 4, 5, or 6 dpi for groups of four female rabbits. The treatment groups have been separated based upon initiation of treatment into pre (−1 to 2 dpi) and post symptomatic (3 to 6 dpi) for comparison of all data. The systemic RPV induced disease (including fever and secondary lesion formation) is not observable until 3–4 dpi and therefore is the rationale for this separation.

All vehicle treated control rabbits required euthanasia on Day 7 post infection due to respiratory failure and severe RPV induced disease. In contrast, all rabbits treated with CMX001 survived for the 14-day study period when treatment was initiated at 4 dpi or earlier (Table 3). When treatment was initiated at 5 dpi, three of four rabbits survived and when treatment was initiated on 6 dpi one of four rabbits survived.

In groups in which CMX001 treatment was initiated at the pre symptomatic stage (−1 to 2 dpi), animals had few disease symptoms including fever, weight loss and average clinical score profiles over the course of the experiment (Figure 8A–C). All animals in the pre symptomatic treatment groups did exhibit a body temperature above 39.5 °C between 4 and 9 dpi and slight weight loss that recovered by 12 dpi for all groups. The primary lesions (Figure 8G top row) were increasing larger as the delay in dosing increased, however were small compared to the vehicle treated animals and all were healed by 14 dpi. There were few secondary lesions observed in groups where treatment was initiated at −1 to 1 dpi, with more secondary lesions present on animals in which treatment was initiated at 2 dpi. No animals exhibited severe respiratory disease during the course of the experiment.

Post symptomatic stage treated groups of animals (treatment initiated at 3 to 6 dpi) exhibited more pronounced RPV induced disease than in the pre symptomatic treatment groups. Animals that received CMX001 treatment beginning at 3 dpi exhibited disease nearly indistinguishable from that of animals receiving CMX001 beginning at 2 dpi, however the primary lesion was larger in size and there were more secondary lesions, as would be expected from a 1 day delay in initiating treatment. While all the animals survived when treatment was begun at 4 dpi, all animals showed numerous secondary lesions and fairly substantial weight loss with a larger primary lesion exhibiting necrosis. The three of four animals that survived from the group in which treatment was initiated on 5 dpi had numerous secondary lesions, weight loss and moderate respiratory disease. When treatment was initiated at 6 dpi all animals exhibited severe RPV disease-related symptoms characterized by large, necrotic primary lesion, numerous secondary lesions, weight loss and respiratory disease (Figure 8D–F). It is important to note that at 5–6 dpi in the course of a normal, untreated RPV infection, the animals are demonstrating severe, advanced disease with fevers, weight loss, numerous secondary lesions and respiratory disease.

Maximum clinical score and weight loss were again used as indications of maximal illness over for all groups tested. It was observed that in groups in which all animals survived (0 to 3 dpi) with few clinical symptoms the maximum average clinical scores were significantly lower than that of the vehicle treated animals (Figure 9A). Animals that began treatment 1 day prior to infection had a lower maximum clinical score than that of vehicle treated animals, it was, however, not significant. There was an advantage noted to having treatment for five days in the presence of virus infection when comparing the group in which treatment was initiated on −1 dpi as compared to those that began treatment 0 to 2 dpi. As treatment was initiated later in infection, as expected the disease severity increased, showing a time of treatment dependence in the maximum disease severity observed. In groups in which not all the animals survived (5 and 6 dpi treatment groups), there was little difference in the maximum clinical score as compared to vehicle treated animals.

As observed with the maximum clinical score, the maximum average weight loss also exhibited a time of treatment initiation dependence on the maximal weight loss. When treatment was begun by 2 dpi animals exhibited little weight loss, while when treatment was begun between 3 and 6 dpi animals exhibited weight loss in excess of the vehicle treated group (Figure 9B). This is likely due to the fact that the vehicle treated animals are euthanized due to severe respiratory disease before significant weight loss can occur.

We found that the initiation of CMX001 treatment can be delayed to 4 dpi for protection against RPV induced morbidity and mortality. There was a statistically significant protection against RPV induced disease when treatment was delayed up to 5 dpi. This allows for a large window for treatment opportunity from pre- to post-exposure and treatment of systemic disease with significant protection and reduction in RPV disease.

## 3. Experimental Section

### 3.1. Cell and Virus Growth

CV-1 cells were maintained in Minimum Essential Media (MEM) with Earle’s Salts (Gibco, New York, USA) supplemented with 2 mM glutamine (Media Tech, Virginia, USA), 50 U/mL penicillin G and 50 μg/mL streptomycin (Media Tech), 1 mM sodium pyruvate (Media Tech), and 0.1 mM nonessential amino acids (Media Tech), and 10% v/v FBS (Gibco).

Rabbitpox virus (RPV) was obtained from ATCC and virus stocks used in these experiments were ≤5 passages from the original stock and have maintained virulence as evidenced by an LD_50_ in 9-week old NZW rabbits of approximately 10 pfu by the intradermal route. All viruses for animal infections were pad purified over 36% sucrose using standard methods and resuspended in PBS. Viruses were titered on CV-1 cells using standard methods. [45,46]

### 3.2. Housing of Animals

For intradermally infected rabbits, 8 week (3–4lb) old NZW rabbits were obtained from Myrtle’s Rabbitry (Thompson Station, TN) and housed in standard in stainless steel solid back and side cages at 20 °C and 12 h light/12 h dark regime. All animals obtained a unique ear tattoo number in the left ear prior to arrival at the study site. Animals were allowed to acclimate to their surroundings for 5–10 days prior to RPV infection. Food and water were available to the animals *ad libitum*. Rabbits received apples, alfalfa cubes, or fresh greens daily for enrichment.

All animal procedures were approved by the University of Florida Institutional Animal Care and Use Committee.

### 3.3. Animal Infections

Intradermal infection was performed by bilateral shaving of both thighs of the rabbit, sterilizing with an isopropanol wipe followed by intradermal injection of 100 or 1000 pfu RPV using a 27 gauge needle [19]. The injection site was then traced using a black permanent marker to note the site of infection.

### 3.4. Monitoring of Animals

Rabbit were microchipped at the time of infection to transmit body temperature and unique identification number (Bio Medic Data Systems, Seaford, DE). Rabbits underwent a complete physical daily. Rabbits were euthanized upon the onset of severe respiratory distress (labored, extremely slow or open mouth breathing) or weight loss of greater than 15% of initial body weight.

Clinical scores for each rabbit each day were generated by assigning numerical values of 0 to 4 for all clinical measurements obtained during the course of the evaluation of each rabbit. These measurements included: weight change (loss, no change or gain), body temperature (low, within normal limits, fever), respiration rate (normal, depressed), heart rate (normal, decreased), intake and output, overall attitude and posture, presence of secondary lesions (number of secondary lesions and number of sites present) and primary site of infection condition (degree of reaction). These scores are used as a measure of disease severity, where the higher the number the more severe the disease.

Weight change was calculated as the percent change from weight on the day of infection with RPV. All statistical analysis was performed using an unpaired t test for each group measurement as compared to vehicle treatment groups.

All animal procedures and euthanasia were carried out according to the University of Florida IACUC guidelines.

### 3.5. CMX001 Dosing of Animals

Animals were trained for 4–5 days prior to the initiation of the study to readily accept the 10% sucrose solution used to deliver CMX001 (HDP-CDV). The 10% sucrose training solution, vehicle solution and CMX001 was administered orally using a 1 mL disposable syringe with the end dipped in granulated sugar and fed orally to the animals. The total volume of liquid the animals received was approximately 1.0 mL. Dry powdered CMX001 (Chimerix Inc, North Carolina, USA) was dissolved in 10% sucrose in water with food coloring to the required concentration. Vehicle treated animals received 10% sucrose solution only. In a previous pharmacokinetic study all rabbits treated with CMX001 for 7 days at doses up to 25 mg/kg/day survived to the scheduled termination. In this special issue there is a review on the development of CMX001 for further toxicological data.

## 4. Conclusions

We reviewed the salient features of the rabbitpox virus infection of rabbits as a model system for systemic orthopoxvirus disease, key features of human smallpox, monkeypox and certain adverse complications arising from vaccination. We then used this system to evaluate the ability of CMX001, either administered prophylactically or after exposure but prior to the onset of symptoms to prevent death and clinical symptoms. Our findings suggest that CMX001 administered either prophylactically or soon after infection prior to the onset of symptoms is very effective in preventing death and lessening clinical symptoms of rabbitpox infection. It is important to note that CMX001 has a mechanism of action different from that of ST-246 [47], another effective smallpox antiviral drug under development. Having multiple antiviral drugs with different mechanisms of action decreases the risk of developing resistance. We believe that the powerful antiviral effects of CMX001 suggest that the drug would be a very effective treatment for human smallpox or other orthopoxvirus infections. Every concentration of CMX001 from 1 mg/kg to 20 mg/kg tested gave significant protection when treatment was began prior to or within 24 hours of infection independent of whether the drug was administered once or twice a day.

Treatment could be delayed up to 4 dpi when administered twice a day at 5 mg/kg for five days and give complete protection from RPV morbidity and mortality. Administration of CMX001 at 5 dpi provided 75% survival in animals evaluated. These results suggest that a strict schedule of dosing is not critical to maintain antiviral effects *in vivo*. It is important to note, perhaps not surprisingly, that even though virtually any treatment prevents death, that depending on dose and delay in onset of treatment, significant clinical symptoms can occur. The severity of symptoms correlates with lower doses and delays in beginning treatment. The delay in treatment post exposure will be examined further in light of results in the final study presented in this paper in the accompanying paper dealing with post symptomatic treatment with CMX001 of RPV infected rabbits.

## Figures and Tables

**Figure 1.**
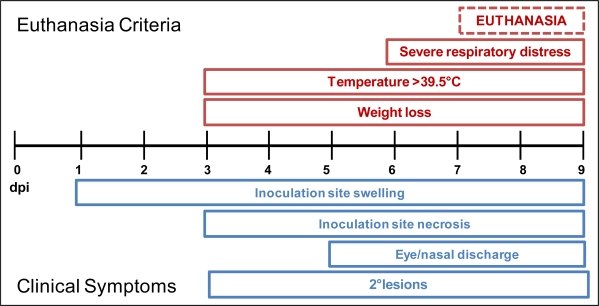
Timeline of disease in 8–10 week old New Zealand White rabbits infected intradermally with 1000 pfu rabbitpox virus (RPV). Symptoms in boxes above the timeline represent clinical measurements that contribute to euthanasia guidelines. Clinical symptoms are located below the timeline. Reproduced with permission from American Society for Microbiology [19].

**Figure 2.**
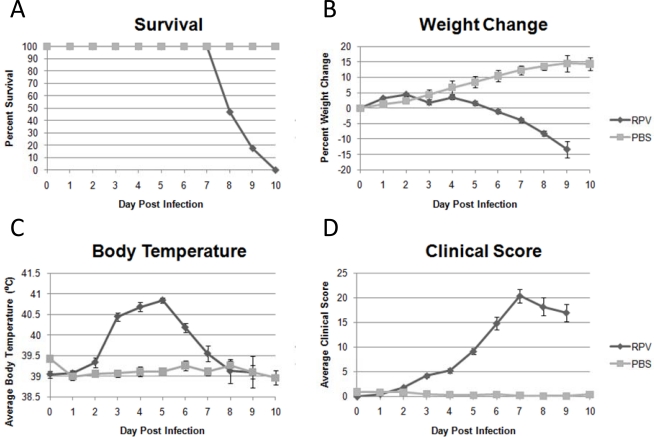
Disease in rabbits lethally infected with RPV compared to age/weight matched mock infected rabbits. (**A**) Survival; (**B**) Average weight change from weight at time of infection for each group. Negative values represent weight loss. (**C**) Average body temperatures over time. Temperatures over 39.5 °C are considered a fever. (**D**) Average clinical score over time. Error bars represent SEM. Reproduced with permission from American Society for Microbiology [19].

**Figure 3.**
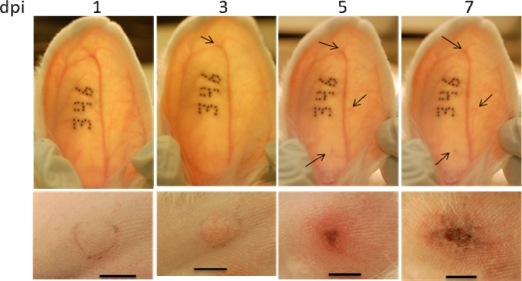
Ears and primary inoculation site of RPV infected NZW rabbits over time. Rabbits were infected as described in Figure 2. The top row of photographs shows the progression of secondary lesions in the ears of RPV infected rabbits. Arrows note the site of secondary lesions. The bottom row of photographs shows the progression of the primary lesions over time. Bars represent 1 cm.

**Figure 4.**
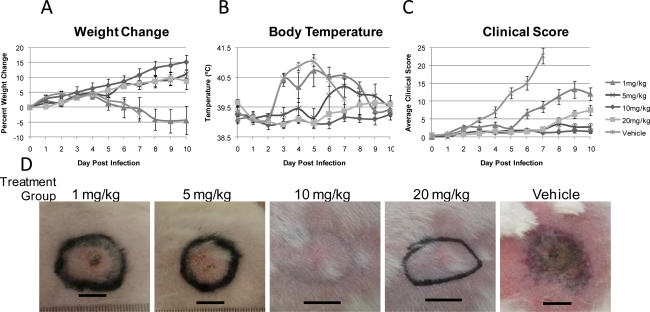
Clinical observations for evaluation of minimum dosage of CMX001 to provide protection from RPV disease. Animals were dosed at concentrations and schedules as outlined in Table 1. (**A**) Average weight change from weight at day of infection. Negative values indicated weight loss. (**B**) Average body temperatures. (**C**) Average clinical scores. (**D**) Pictures of primary lesions from representative animals at 7 dpi. Bars represent 1 cm. Black circles denote site of intradermal inoculation.

**Figure 5.**
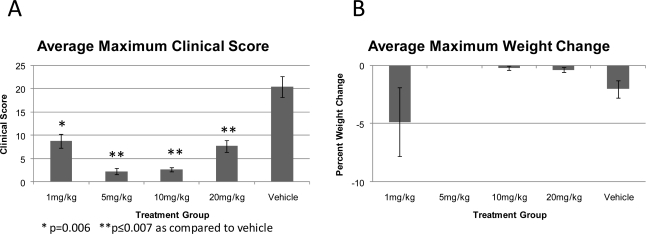
Disease severity measurements for evaluation of minimum dosage of CMX001 to provide protection from RPV disease. Animals were dosed at concentrations and schedules as outlined in Table 1. (**A**) Average maximum clinical scores for each animal per group over the course of the experiment. (**B**) Average maximum percent weight loss from weight at day 0 for each animal per group over the course of the experiment.

**Figure 6.**
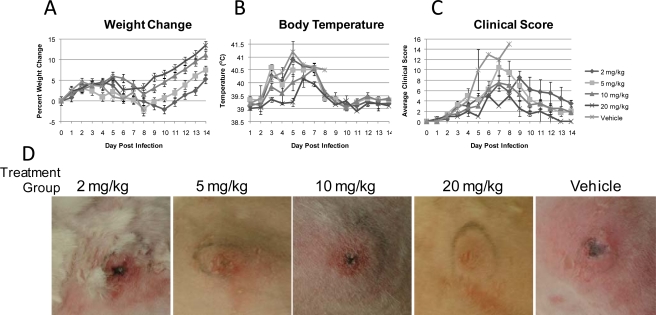
Clinical observations for evaluation of minimum dose of CMX001 required for protection from RPV when treatment is begun 1 day post infection. Animals were dosed at concentrations and schedules as outlined in Table 2. (**A**) Average weight change for each group. (**B**) Average body temperatures for each group. (**C**) Average clinical scores for each group. Error bars represent SEM. (**D**) Representative photos of primary lesions for treatment groups at 5 dpi.

**Figure 7.**
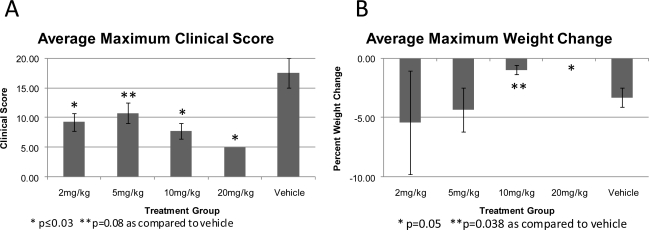
Disease severity measurements for evaluation of minimum dose of CMX001 required for protection from RPV when treatment is begun 1 day post infection. Animals were dosed at concentrations and schedules as outlined in Table 2. (**A**) Average maximum clinical scores for each animal per group over the course of the experiment. (**B**) Average maximum percent weight loss from weight at day 0 for each animal per group over the course of the experiment.

**Figure 8.**
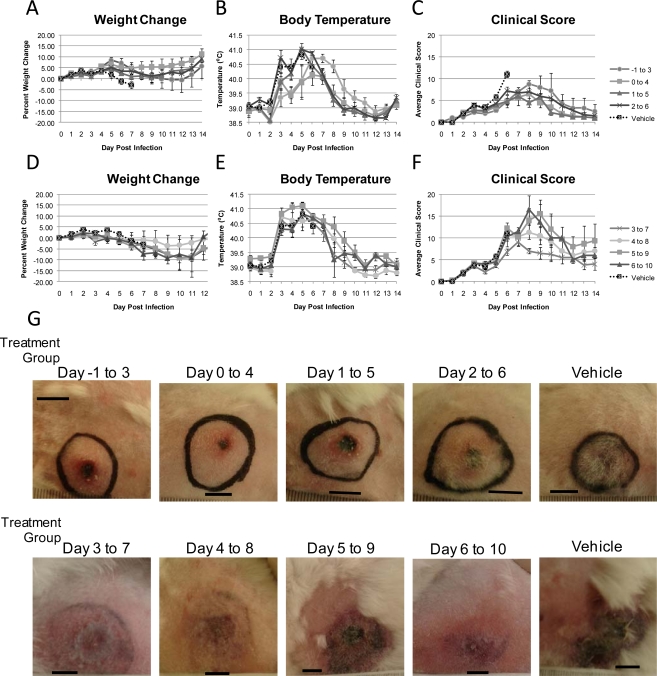
Clinical observations for evaluation of survival when dosage with CMX001 is delayed up to six days post infection. Animals were dosed at concentrations and schedules as outlined in Table 3. (**A**) Average weight change from weight at day of infection. Negative values indicated weight loss (**B**) Average body temperatures. (**C**) Average clinical scores. The higher the number, the more severe the disease presentation. (**D**) Pictures of primary lesions from representative animals at 6 or 7 dpi. Bars represent 1 cm. Black circles denote site of intradermal inoculation.

**Figure 9.**
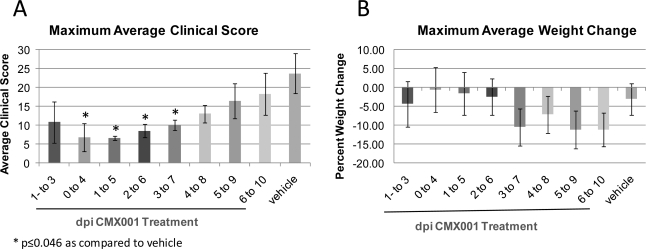
Disease severity measurements for evaluation of survival when dosage with CMX001 is delayed up to six days post infection. Animals were dosed at concentrations and schedules as outlined in Table 3. (**A**) Average of maximum clinical scores for each animal per group over the course of the experiment. (**B**) Average of maximum percent weight loss from weight at day 0 for each animal per group over the course of the experiment.

**Table 1.** Minimum dosage of orally administered CMX001 that provides protection to RPV disease when treatment is begun one day prior to infection with RPV. QD designates drug administered once a day; BID designates drug administered twice a day at concentrations indicated in the table.

**CMX001 Dose (mg/kg)**	**Dosing Frequency**	**Day of Dosing (dpi)**	**Mean Time to Death ± SEM**	**Survival at Day 14PI**
1	BID	−1 to 3	NA	4/4 (100%)*
5	BID	−1 to 3	NA	4/4 (100%)*
10	BID	−1 to 3	NA	6/6 (100%)**
20	QD	−1 to 3	NA	6/6 (100%)**
Vehicle	BID	−1 to 3	6.6 ± 0.16	0/10 (0%)

* p = 0.0099;

** p = 0.0012 as compared to vehicle.

**Table 2.** Evaluation of minimal dosage of CMX001 that provides protection when administered once a day (QD) when dosing is begun 1 day post infection for five days.

**CMX001 Dose (mg/kg)**	**Dosing Frequency**	**Day of Dosing (dpi)**	**Mean Time to Death ± SEM**	**Survival at Day 14PI**
2	QD	1 to 5	NA	4/4 (100%)*
5	QD	1 to 5	NA	4/4 (100%)*
10	QD	1 to 5	NA	4/4 (100%)*
20	QD	1 to 5	NA	2/2 (100%)**
Vehicle	QD	1 to 5	7±1	0/2 (0%)

* p = 0.06;

** p = 0.33 as compared to vehicle.

**Table 3.** Evaluation of survival when dosage with CMX001 is delayed up to 6 days post infection. Animals were dosed twice a day (BID) for 5 days beginning at day indicated in the days of dosing column of the table. All animals received 5mg/kg CMX0001.

**CMX001 Dose (mg/kg)**	**Frequency of Dosing**	**Day of Dosing (dpi)**	**Mean Time to Death ± SEM**	**Survival at Day 14PI**
5	BID	−1 to 3	NA	4/4 (100%)
5	BID	0 to 4	NA	4/4 (100%)
5	BID	1 to 5	NA	4/4 (100%)
5	BID	2 to 6	NA	4/4 (100%)
5	BID	3 to 7	NA	4/4 (100%)
5	BID	4 to 8	NA	4/4 (100%)
5	BID	5 to 9	9 ± 0	3/4 (75%)
5	BID	6 to 10	9.3 ± 0.9	1/4 (25%)
Vehicle	BID	Vehicle	7 ± 0	0/4 (0%)
